# Familial Pancreatic Cancer: To Screen or not to Screen?

**DOI:** 10.1016/j.ebiom.2015.12.014

**Published:** 2015-12-17

**Authors:** Hans F.A. Vasen, Detlef Bartsch

**Affiliations:** a Department of Gastroenterology & Hepatology, Leiden University Medical Center, Leiden, The Netherlands; b Department of Visceral-, Thoracic- and Vascular Surgery, University Hospital Marburg, Marburg, Germany

In this issue of *EBioMedicine*, Pandharipande and colleagues report important data that may well assist the decision-making process when screening families at risk for pancreatic ductal adenocarcinoma (PDAC) (Pandharipande et al., 2015). The aim of the study was to compare, using a simulation model, the effectiveness of different screening strategies in *BRCA2*-mutation carriers.

Hereditary factors play a role in the development of PDAC in about 5% of all cases (Bartsch et al., 2012). Individuals at increased risk of developing PDAC can be subdivided into those with an underlying gene defect such as *CDKN2A*, *BRCA1*/*2*, *PALB2* and *STK11*-mutations and those individuals with a significant family history of PDAC (Familial pancreatic cancer (FPC)). Carriers of a *BRCA2* mutation constitute the largest group of mutation carriers at risk for PDAC. The risk of developing PDAC in this group is between 5–10%, depending on the number of patients with PDAC in the family (van Asperen et al., 2005).

The prognosis of patients with PDAC has not meaningfully improved over the recent decades and it is now clear that the only way to improve matters will be through detection of early stage PDAC, or even better its precursor lesions (IPMN or PanIN3). Interest in screening individuals at risk for PDAC has also increased over the last decade.

An international consortium of experts recently recommended that screening should be considered in individuals with a > 5% lifetime risk of PDAC (Canto et al., 2013). However, studies published so far (mostly on FPC) have produced rather disappointing outcomes. Besides the very low screening yield (PDAC in 1–2%), the majority of screen-detected cancers proved to be advanced tumors. A small proportion of patients underwent surgery for precursor lesions, but only a few had high-risk precursor lesions (PanIN3 or high-grade IPMN) (Bartsch et al., 2012).

A major problem that must be confronted when screening individuals at high-risk of PDAC is the potential morbidity (up to 40%) (Keck et al., 2015, Diener et al., 2011) and mortality (0.5–6%) associated with the surgical treatment of suspicious lesions (Keck et al., 2015, Diener et al., 2011), even in specialized centers. Due to a lack of preoperative certainty in many cases as to the nature of the lesion (low or high-risk precursor lesion or cancer), theoretically a patient could die following surgery for a benign, low-risk lesion. Screening may therefore facilitate both the detection of early cancer and an improved prognosis, but at the same time represent a danger due to the inherent complications of surgery. Careful selection of high risk groups prior to enrollment in surveillance program must therefore be a priority.

In the absence of long-term clinical studies, the study by Pandharipande and colleagues provides important information on the effectiveness of screening in relation to the risk of developing PDAC in *BRCA2* mutation carriers (Pandharipande et al., 2015). The authors developed a simulation screening model based on MRI-scanning for families with a *BRCA2* mutation. All available data on cancer risks in mutation carriers, and SEER data on PDAC were used to develop the model. The authors then calculated the expected gain in life expectancy (LE) for different phenotypes and different surveillance protocols, compared to *no screening*. Furthermore, in the sensitivity analysis they evaluated additional screening strategies, screening intervals and surgical mortality.

They found that *one-time* screening of the primary *BRCA2* cohort at age 50 resulted in small LE gains (a few days). *Annual* screening from age 50 was most effective for *BRCA2* mutation carriers with two first-degree relative (FDR) with PDAC (LE gain of 20 days), and for those with three or more FDR with PDAC (260 days) but not for the primary cohort (reduced LE of 13 days). The authors attributed the reduced life expectancy in the latter group to frequent screening in low-risk mutation carriers, as false positive findings and an increased discovery of insignificant lesions represents an increased burden due to the resulting surgical interventions.

Another important finding was that the effectiveness of screening was strongly influenced by the mortality rate associated with the surgical intervention. The gain of LE of *one-time* screening disappeared with a slight increase of the surgical mortality rate to > 2.3%.

A limitation of the study was that data on PDAC risk are not available for individuals with both a *BRCA2* mutation and various numbers of relatives with PDAC.

In conclusion, this study provides important information that could be helpful in the management of individuals at high-risk for PDAC. Although the results of a simulation model study cannot be directly translated to clinical practice, the present study suggests that surveillance of *BRCA2* mutation carriers should probably be restricted to those with the highest risks, i.e., mutation carriers with two or more FDR with PDAC. Moreover, surveillance and management of high-risk groups should only be performed in high-volume expert centers.

Perhaps the most important conclusion is that, before entering a program, all individuals should be clearly informed about the advantages and disadvantages (and dangers) of surveillance. We also suggest that similar studies should now be performed for other syndromes associated with an increased risk of PDAC.
